# Proteomic analysis of protein interactions between *Eimeria maxima* sporozoites and chicken jejunal epithelial cells by shotgun LC-MS/MS

**DOI:** 10.1186/s13071-018-2818-4

**Published:** 2018-04-04

**Authors:** Jingwei Huang, Tingqi Liu, Ke Li, Xiaokai Song, Ruofeng Yan, Lixin Xu, Xiangrui Li

**Affiliations:** College of Veterinary Medicine, Nanjing Agricultural University, 1 Weigang, Nanjing, Jiangsu 210095 People’s Republic of China

**Keywords:** *Eimeria maxima*, Invasion, Chicken, Jejunal epithelial cells, Parasite-host interaction

## Abstract

**Background:**

*Eimeria maxima* initiates infection by invading the jejunal epithelial cells of chicken. However, the proteins involved in invasion remain unknown. The research of the molecules that participate in the interactions between *E. maxima* sporozoites and host target cells will fill a gap in our understanding of the invasion system of this parasitic pathogen.

**Methods:**

In the present study, chicken jejunal epithelial cells were isolated and cultured *in vitro*. Western blot was employed to analyze the soluble proteins of *E. maxima* sporozoites that bound to chicken jejunal epithelial cells. Co-immunoprecipitation (co-IP) assay was used to separate the *E. maxima* proteins that bound to chicken jejunal epithelial cells. Shotgun LC-MS/MS technique was used for proteomics identification and Gene Ontology was employed for the bioinformatics analysis.

**Results:**

The results of Western blot analysis showed that four proteins bands from jejunal epithelial cells co-cultured with soluble proteins of *E. maxima* sporozoites were recognized by the positive sera, with molecular weights of 70, 90, 95 and 130 kDa. The co-IP dilutions were analyzed by shotgun LC-MS/MS. A total of 204 proteins were identified in the *E. maxima* protein database using the MASCOT search engine. Thirty-five proteins including microneme protein 3 and 7 had more than two unique peptide counts and were annotated using Gene Ontology for molecular function, biological process and cellular localization. The results revealed that of the 35 annotated peptides, 22 (62.86%) were associated with binding activity and 15 (42.86%) were involved in catalytic activity.

**Conclusions:**

Our findings provide an insight into the interaction between *E. maxima* and the corresponding host cells and it is important for the understanding of molecular mechanisms underlying *E. maxima* invasion.

**Electronic supplementary material:**

The online version of this article (10.1186/s13071-018-2818-4) contains supplementary material, which is available to authorized users.

## Background

Coccidiosis is an enteric disease, caused by the unicellular parasite *Eimeria*, and a major problem in the poultry industry [1]. *Eimeria maxima* is one of the most prevalent *Eimeria* species that causes substantial economic losses worldwide [2]. *Eimeria maxima* undergoes schizogony and gametogony after the invasion of jejunal epithelial cells and subsequently occupies the cell cytoplasm [3]. Although an *in vitro* model has been developed to study the invasion of Madin-Darby bovine kidney (MDBK) cells by sporozoites [4], the mechanisms by which infective sporozoites and merozoites recognize, invade and migrate within the intestinal epithelium remain unknown. However, the establishment of *E. maxima* in the appropriate habitat is important in host infection.

Host-cell invasion by apicomplexan parasites is a unique process dependent on gliding motility and requires a transmembrane link between the parasite cytoskeleton and the host cell [5]. Many proteins are secreted by the parasite to help it penetrate the defense barriers, invade the host cells and avoid the immune attack of the host [6]. An event shared by these processes is the appropriate engagement and disengagement of specific receptors upon the regulated apical release of invasion proteins from parasite secretory organelles (micronemes, rhoptries) [7]. Therefore, the identification of soluble protein components of the invasive sporozoite stage functioning at the interface between *E. maxima* and host intestinal epithelial cells is crucial to understand the mechanism of parasite invasion and identify possible vaccine target.

In this study, Western blot, co-IP, and shotgun LC-MS/MS were combined to characterize *E. maxima* sporozoite proteins binding to the chicken jejunal epithelial cells and to screen the proteins related to invasion.

## Methods

### Parasites and animals

The parasites *E. maxima* and Chinese yellow chickens used in this study were obtained and kept in conditions as previously described [8].

### Collection and purification of sporozoites

Sporozoites from *E. maxima* sporulated oocysts were purified using DE-52 anion exchange columns (GE Whatman, Pleasanton, USA) according to a protocol described previously [9]. Finally, the collected sporozoites were stored in liquid nitrogen before use.

### Soluble antigens of *E. maxima*

The soluble proteins of *E. maxima* were prepared following a protocol described previously with slight modifications [2]. Briefly, the pellet of 5 × 10^8^ sporozoites was dissolved in 2 ml of PBS containing 0.5% Triton X-100 and 1% protease inhibitor cocktail set III (Millipore, Burlington, USA) and was disrupted by ultrasound in an ice bath. Then, the supernatant proteins were separated by high-speed centrifugation (14,000× *g*, 30 min, 4 °C), aliquoted and stored at -70 °C until used in co-culture with chicken jejunal epithelial cells. Twenty micrograms of *E. maxima* soluble proteins were subjected to SDS-PAGE analysis using a 12% gel.

### Antisera against soluble proteins of *E. maxima*

The polyclonal sera against *E. maxima* soluble proteins were prepared according to a method described previously [10]. Briefly, approximately 100 μg of soluble proteins of *E. maxima* sporozoites were mixed with Freund’s complete adjuvant (Invitrogen, Carlsbad, USA) and injected intraperitoneally into naïve SD rats (two-week-old, female). After a two-week interval, 100 μg of soluble proteins of *E. maxima* sporozoites in Freund’s incomplete adjuvant (Invitrogen) were intraperitoneally injected. In total three boosters were conducted. The serum was collected 14 days after the last injection and stored at -70 °C until use. In addition, sera collected before proteins injection was used as a negative control.

### Isolation of chicken jejunal epithelial cells

Chicken jejunal epithelial cells were isolated using a method described previously [11, 12]. Coccidian-free chickens were immerged for 5 min in 70% ethanol after they were slaughtered and the blood was drained. After that, the jejunums were dislodged and put into Hanks’ balanced salt solution (HBSS, PAA, Linz, Austria). Then the jejunums were washed by HBSS until the mucus was completely removed. Following dissection of the mucosa into small strips, the strips were put into 1mM DTT (Sigma-Aldrich, St. Louis, USA) in 50 HBSS (30 min at ambient temperature). Sequentially, the mucosal strips were incubated in 1 mM EDTA (Sigma) for 10 min at 37 °C. Mucosal strips were briefly rinsed in HBSS (to eliminate already detached jejunal epithelial cells), transferred to fresh HBSS at ambient temperature and followed by 5–10 vigorous shakes of the container. This procedure leads to instant detachment of jejunal epithelial cells in a full-length crypt formation. After rapid removal of the mucosal strips by passing the solution over a coarse mesh (80 μm, Rotilabo sieve, Carl Roth GmbH, Karlsruhe, Germany), rapid purification of detached jejuna epithelial cells was achieved by usage of a mesh filter (400 μm pore size, Sefer, Kansas City, KS, USA) fixed with tape to a plastic ring (5 cm diameter, 2 cm height and 3 mm thickness). The suspension containing jejunal epithelial cells crypts was gently but rapidly passed over the mesh to separate the jejunal epithelial cells crypts from single cells (erythrocytes, leukocytes, fibroblast, etc.) which easily passed through the filter. The filter was then rapidly inverted and purified intact jejuna epithelial cells crypts were immediately washed out with culture medium (see below) at ambient temperature. The jejunal epithelial cells crypt solution was then rapidly transferred to the ECM-coated culture dish and cultured at 41 °C and CO_2_ for 1.5 h. The non-adherent cells were collected for identification of the jejunal epithelial cells and co-culture with the soluble proteins of E. maxima sporozoite.

Co-culture of jejunal epithelial cells and soluble proteins of E. maxima sporozoites

The collected jejunal epithelial cells were suspended in an advanced culture medium [DMEM containing 5% fetal bovine serum (FBS), 100 U/ml penicillin and 100 mg/ml streptomycin, 20 mmol/l HEPES, 1 mmol/l Supplement, 5 mg/l bovine pituitary extract, and 0.01 mg/l epidermal growth factor (EGF) (all products were from Gibco Life technologies, New York, USA)]. Subsequently, the cells were seeded in a 6-well plate (Corning, Corning, NY, USA; 1 × 10^6^ cells per well) and cultured at 41 °C in an atmosphere of 95% air and 5% CO_2_ for 2 h. Following sterilization using a 0.22 μm filter (Millipore), the soluble proteins (final concentration of 100 μg/ml) of E. maxima sporozoites were added to the culture medium and incubated for 1 h. The jejunal epithelial cells were washed four times with 0.1 M PBS buffer (pH 7.8) after removal of the culture medium.

### Extraction of proteins from co-cultured jejunal epithelial cells

The washed cells were mixed with 0.2 ml cell lysis (pH 7.2, 60 mM Tris-HCl; 2% sodium dodecyl sulfate; SDS) containing 1% protease inhibitor cocktail set III (Millipore), subjected to three freeze-thaw cycles at -80 °C and 37 °C, and sonicated in an ice bath as described above. The soluble supernatant was filtered through a 0.22 μm mesh filter, aliquoted and stored at -70 °C before use.

### SDS-PAGE and Western blot analysis

Twenty micrograms of protein samples from co-cultured jejunal epithelial cells were subjected to SDS-PAGE using a routine method described elsewhere [13].

Western blot was employed to analyze the soluble proteins of *E. maxima* sporozoite that bound to chicken jejunal epithelial cells. Briefly, the protein samples were separated using a 12% SDS-PAGE gel as described above and transferred to a nitrocellulose membrane (Millipore) using Semi-Dry Transfer Cell (Bio-Rad, Hercules, USA) according to the manufacturer’s instructions. Rat antiserum against the soluble proteins of *E. maxima* sporozoite (dilution 1:200) was used as the primary antibody and horseradish peroxidase (HRP)-conjugated goat anti-rat IgG (dilution 1:2000, Sigma) was used as the secondary antibody. The bound antibodies were detected using Pierce DAB Substrate Kit (Thermo Fisher Scientific, Waltham, USA) according to the manufacturer’s instructions.

### Co-immunoprecipitation (co-IP) assay

The co-IP assay was conducted according to the manufacturer’s instructions (Protein A/G PLUS-Agarose: sc-2003, Santa Cruz Biotechnology Inc., Delaware Ave Santa Cruz, USA). Briefly, 5 × 10^7^ washed jejunal epithelial cells were incubated with 3 ml of ice-cold RIPA buffer (Vazyme Biotech Co. Ltd., Nanjing, China) containing 1% protease inhibitor cocktail set III (Millipore) for 15 min on ice and were disrupted by repeated passage through a 21-gauge needle. Cellular debris was removed by centrifugation at 13,000× *g* for 10 min at 4 °C, and the supernatant was transferred to a microcentrifuge tube on ice. One microgram of normal rat polyclonal IgG and 20 μl of suspended Protein A/G-Agarose (25%, V/V) was added to the supernatant and incubated at 4 °C for 1 h on a rotator. The supernatant was separated by centrifugation at 1,000× *g* for 5 min at 4 °C and transferred to a microcentrifuge tube on ice. Two micrograms of the primary antibody was added to the supernatant and incubated at 4 °C for 2 h. Twenty microliters of the suspended Protein A/G-Agarose (25%, V/V) was added to the supernatant and incubated at 4 °C overnight. The immunoprecipitates were collected by centrifugation at 1,000× *g* for 5 min at 4 °C and the supernatant was carefully aspirated and discarded. The immunoprecipitates were gently washed four times with 1 ml of ice-cold RIPA buffer (Vazyme biotech Co. Ltd.) containing 1% protease inhibitor cocktail set III (Millipore). After the final wash, 40 μl of 2× electrophoresis loading buffer (Vazyme biotech Co. Ltd.) was used to resuspend the pellet. The immunoprecipitated samples were boiled for 2–3 min and stored at -20 °C for protein identification.

### Liquid chromatography and tandem mass spectrometry

The immunoprecipitates were sent to Applied Protein Technology Co. Ltd. (Shanghai, China) and the assays were conducted on a Q Exactive mass spectrometer coupled to Easy nLC (Thermo Fisher Scientific) using a routine method [14]. Briefly, trypsin-digested peptides (approximately 30 μg) were trapped and desalted on Zorbax 300SB-C18 peptide traps (Agilent Technologies, Wilmington, DE, USA) and separated on a C18-reversed phase column (0.15 × 150 mm, Column Technology Inc., Fremont, CA, USA). The Easy nLC system (Thermo Fisher Scientific) was used to deliver mobile phases A (0.1% formic acid in HPLC-grade water) and B (0.1% formic acid in 84% acetonitrile) with a linear gradient of 4–50% B (50 min), 50–100% B (4 min), and then 100% B (6 min) at a flow rate of 250 nl/min. To acquire the MS data, a data-dependent top ten method was used, in which the ten most abundant precursor ions were selected for HCD fragmentation.

### Protein identification and annotation

MS/MS spectra were searched using MASCOT 2.2 (Matrix Science, London, UK) against the non-redundant International Protein Index *Eimeria maxima* sequence database version 3.85 (released at May 2014; 6127 sequences) from the European Bioinformatics Institute (http://www.ebi.ac.uk/). The following parameters were used for protein identification: peptide mass tolerance, 20 ppm; MS/MS tolerance, 0.1 Da; enzyme, trypsin; number of missed cleavages, 2; fixed modification, carbamidomethyl (C); variable modification, oxidation (M); score > 20.

### InterProScan annotation and Gene Ontology (GO) categories

InterProScan software (http://www.ebi.ac.uk/interpro/search/sequence-search) was used to perform protein sequences searches against InterPro databases and identify protein signatures [15]. The matched terms were output in RAW format. The RAW data were subjected to GO categories using the Web Gene Ontology Annotation Plot (WEGO) [16]. The three groups of datasets were simultaneously subjected to online analysis (http://wego.genomics.org.cn/) and were compared in one graph. The *P*-values were calculated using the Pearson’s Chi-square test.

## Results

### SDS-PAGE and Western blot analysis of soluble proteins of *E. maxima* sporozoites

**Fig. 1:**
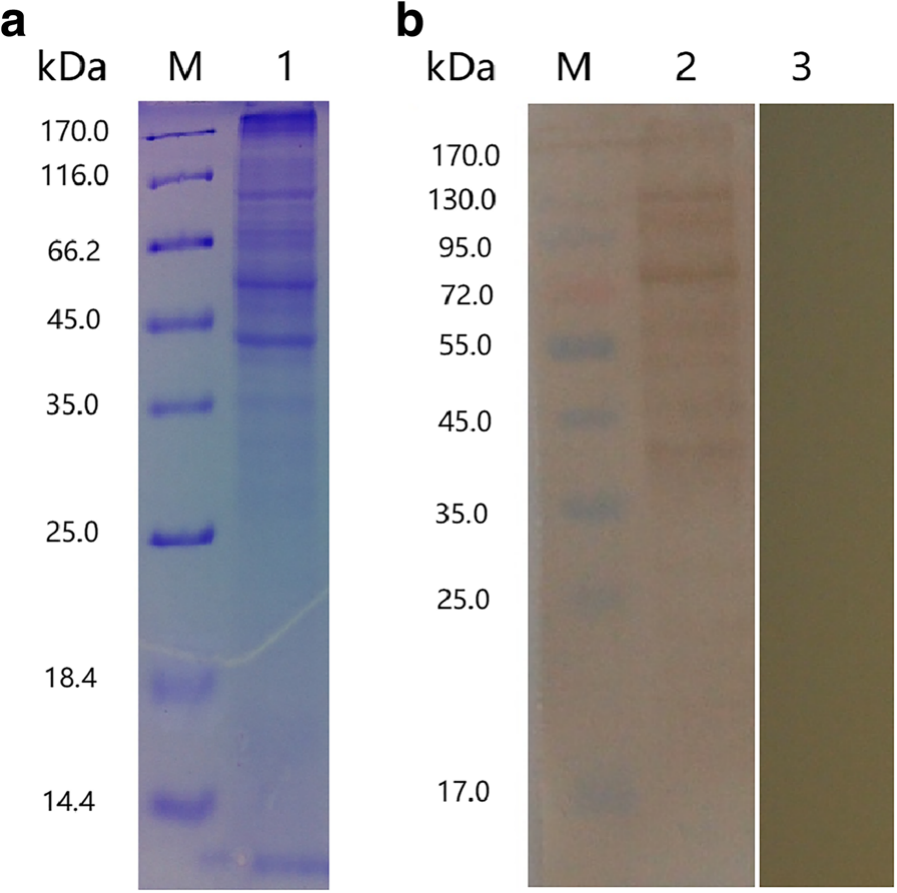
**a** SDS-PAGE analysis of the soluble proteins of *E. maxima* sporozoites. Twenty micrograms of protein samples were subjected to a 12% gel. Lane M: standard protein molecular marker; Lane 1: the soluble proteins of *E. maxima* sporozoites. **b** Western blot analysis of the soluble proteins of *E. maxima* sporozoites. Proteins are recognized by sera from rats as primary antibody. Lane M: standard protein molecular marker; Lane 2: sera from rat immunized with the soluble proteins of *E. maxima* sporozoites; Lane 3: pre-immune rat sera

The soluble proteins of *E. maxima* sporozoites were analyzed by SDS-PAGE (Fig. 1a). The molecular weight of the protein bands ranged from 10 to 170 kDa.

Western blot analysis indicated that the soluble proteins were identified by the antisera of rats experimentally immunized with the parasite proteins (Fig. 1b). However, these soluble proteins could not be identified by the pre-immune rat sera.

### SDS-PAGE and Western blot analysis of proteins of co-cultured cells

**Fig. 2:**
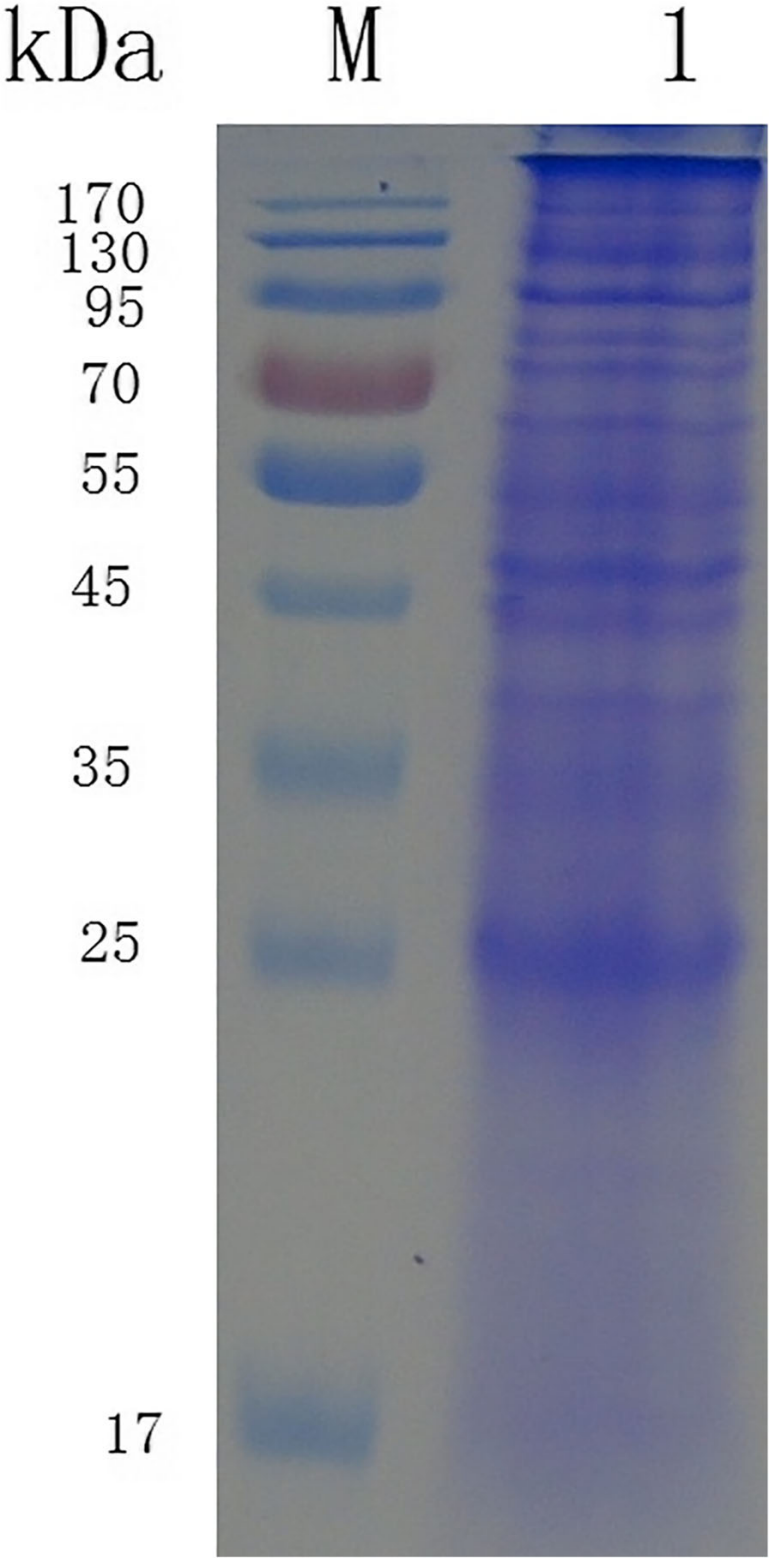
SDS-PAGE analysis of the proteins of chicken jejunal epithelial cells co-cultured with the soluble proteins of *E. maxima* sporozoites. Twenty micrograms of protein samples were subjected to a 12% gel. Lane M: standard protein molecular marker; Lane 1: the protein samples from jejunal epithelial cells co-cultured with the soluble protein of *E. maxima* sporozoites

The fractionated proteins of jejunal epithelial cells co-cultured with soluble proteins of *E. maxima* sporozoites were analyzed by SDS-PAGE and stained with Coomassie brilliant blue. The molecular weight of the protein bands ranged from 17 to 170 kDa (Fig. 2).

**Fig. 3:**
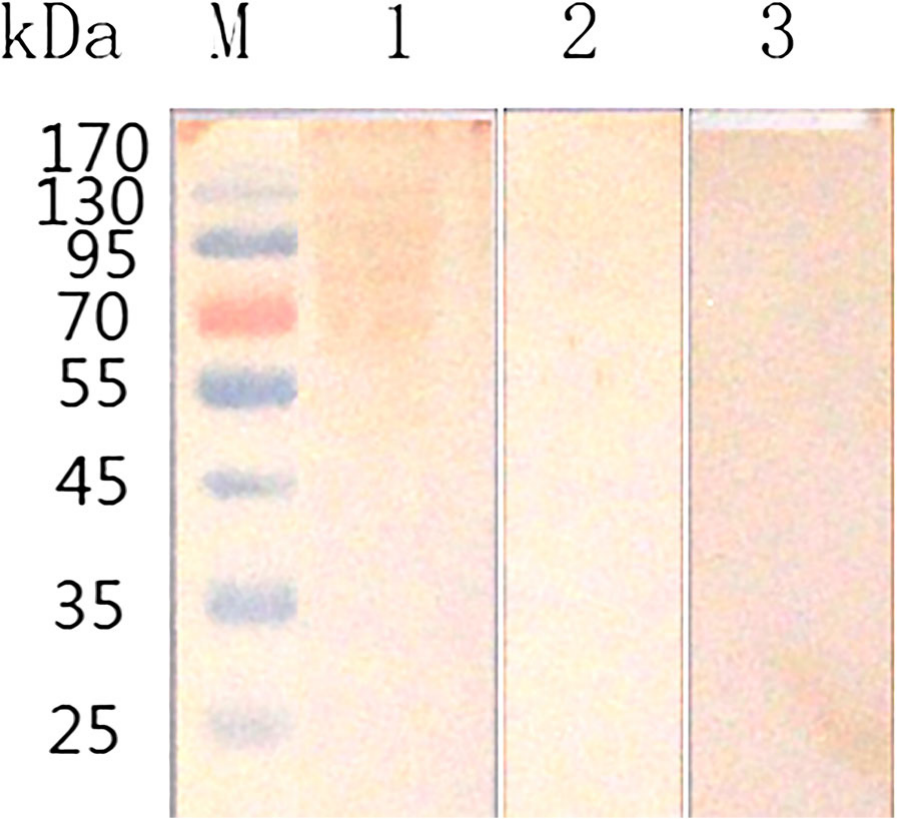
Western blot analysis of proteins of chicken jejunal epithelial cells co-cultured with the soluble proteins of *E. maxima* sporozoites. Proteins are recognized by sera from rats as primary antibody. Lane M: standard protein marker; Lane 1: lysis of chicken jejunal epithelial cells cultured with *E. maxima* sporozoites soluble proteins detected by sera from rats immunized with soluble proteins of *E. maxima* sporozoites; Lane 2: lysis of chicken jejunal epithelial cells cultured with PBS buffer detected by sera from rats immunized with soluble proteins of *E. maxima* sporozoites; Lane 3: lysis of chicken jejunal epithelial cells cultured with *E. maxima* sporozoites soluble proteins detected by pre-immune rat sera

Western blot analysis indicated that rats were successfully seroconverted after experimentally immunized with soluble proteins of *E. maxima* sporozoites. However, no protein bands were detected using pre-immune rat sera. On the other hand, proteins of jejunal epithelial cells co-cultured with PBS buffer as negative control were not able to be detected by antisera of rats immunized with the target proteins. Four protein bands were recognized by the antisera, with molecular weights of 70, 90, 95 and 130 kDa (Fig. 3).

### LC-MS/MS analysis of proteins of *E. maxima* sporozoites binding to jejunal epithelial cells

After analysis by QE shotgun LC-MS/MS [17], 204 non-redundant proteins were identified by searching the *E. maxima* database in Uniprot and NCBI (Additional file 1: Table S1). Only 35 proteins contained more than two unique peptide counts (Additional file 2: Table S2). Among these, microneme proteins (MIC2, MIC3 and MIC7), aldolase, and elongation factors were associated with invasion; 56 and 82 kDa gametocyte antigens were involved in the formation of the oocyst wall and development of the parasite life-cycle, and actin and tubulin were related to parasite movement. Other proteins were involved in cell signaling, including 14-3-3 superfamily proteins. Some of the identified proteins were related to the metabolism of phosphate, lipids, and nucleic acids, including aldolase, enolase 2, RAS small GTPase, and the alpha and beta chains of ATP synthase. Moreover, potential housekeeping genes were significantly enriched, including actin, tubulin, histone, and heat shock proteins. Three hypothetical proteins that had not been characterized were also present in this analysis (Table 1).

**Table 1:** Proteins of *E. maxima* that bound to chicken jejunal epithelial cells identified by shotgun LC-MS/MS with more than two unique peptide counts

Protein description	Accession number	Unique pep count MH+	Pep count (sequence)	Cover percent (%)	Theor MW (kDa)	Theor pI	MASCOT score
Lactate dehydrogenase	gi|557194155	4	4	16.97	35.94	6.60	60.84
14-3-3 protein	gi|557193108	6	6	23.43	32.55	4.80	63.59
Elongation factor 1-alpha	gi|557198794	3	4	6.44	49.36	9.16	63.00
Elongation factor 2	gi|557157066	3	3	3.37	93.28	5.99	49.12
Fructose-bisphosphate aldolase	gi|557193254	3	3	13.13	38.77	8.33	62.10
ATP synthase beta chain	gi|557185041	3	3	4.80	72.32	6.22	47.06
18 kda cyclophilin	gi|557198725	3	3	23.28	20.35	6.97	54.77
Hypothetical protein, conserved	gi|557206977	3	3	11.67	32.25	4.58	53.47
Heat-shock protein 60	gi|557243650	3	3	8.73	59.93	5.86	53.46
B-block-binding subunit of tfiiic protein	gi|557157037	2	9	0.46	34.63	6.76	44.91
Polyubiquitin	gi|557209304	2	5	11.54	17.46	8.01	39.48
RAS small GTpase	gi|557167708	2	3	8.33	22.94	7.64	71.94
Heat-shock protein, related	gi|557184708	5	5	7.65	71.41	5.16	80.62
Microneme protein 7	gi|343094702	2	2	15.12	18.24	4.62	57.25
Tubulin beta chain	gi|343480759	2	2	6.01	49.92	4.78	76.11
ATP synthase alpha	gi|557156948	2	2	6.90	21.68	7.63	43.05
SWI/SNF-related matrix-associated actin-dependent regulator of chromatin	gi|557158395	2	2	1.51	11.91	5.64	41.06
ADP ribosylation factor 1	gi|557167614	2	2	11.40	20.80	6.10	56.12
Protein emb isoform, related	gi|557184804	2	2	0.61	23.58	5.80	35.63
Hypothetical protein, conserved	gi|557168315	2	2	0.38	38.08	6.14	29.12
Hypothetical protein, conserved	gi|557210462	2	2	0.26	63.97	6.65	34.58
60s ribosomal protein L3	gi|557210678	2	2	4.90	44.11	10.41	60.01
Heat-shock protein 90	gi|557188011	2	2	2.94	82.69	5.08	76.54
Microneme protein 2	gi|334851460	5	5	14.64	30.60	4.90	51.92
Heat-shock protein 70	gi|557232143	2	2	3.25	56.17	8.69	46.71
Triosephosphate isomerase	gi|557232104	2	2	10.76	27.36	6.00	38.99
Tubulin alpha chain	gi|557210668	2	2	4.98	46.69	4.90	55.07
Microneme protein 3, partial	gi|557237363	2	2	4.58	44.33	4.65	51.70
Coiled-coil domain-containing protein, related	gi|557238523	2	2	0.45	26.90	4.52	26.72
Actin	gi|557207008	4	15	12.77	42.01	5.00	49.73
Histone	gi|19880141	4	9	20.59	15.39	11.29	35.08
82 kda gametocyte, related	gi|38565038	4	5	9.73	66.09	5.16	101.05
56 kda gametocyte, related	gi|557167757	4	5	9.03	53.30	5.00	78.91
Enolase 2	gi|557157196	4	4	9.37	49.88	5.92	42.82
Glyceraldehyde-3-phosphate dehydrogenase	gi|557168185	4	4	11.21	36.26	7.58	74.56

### Functional categories of proteins of *E. maxima* sporozoites binding to jejunal epithelial cells

**Fig. 4:**
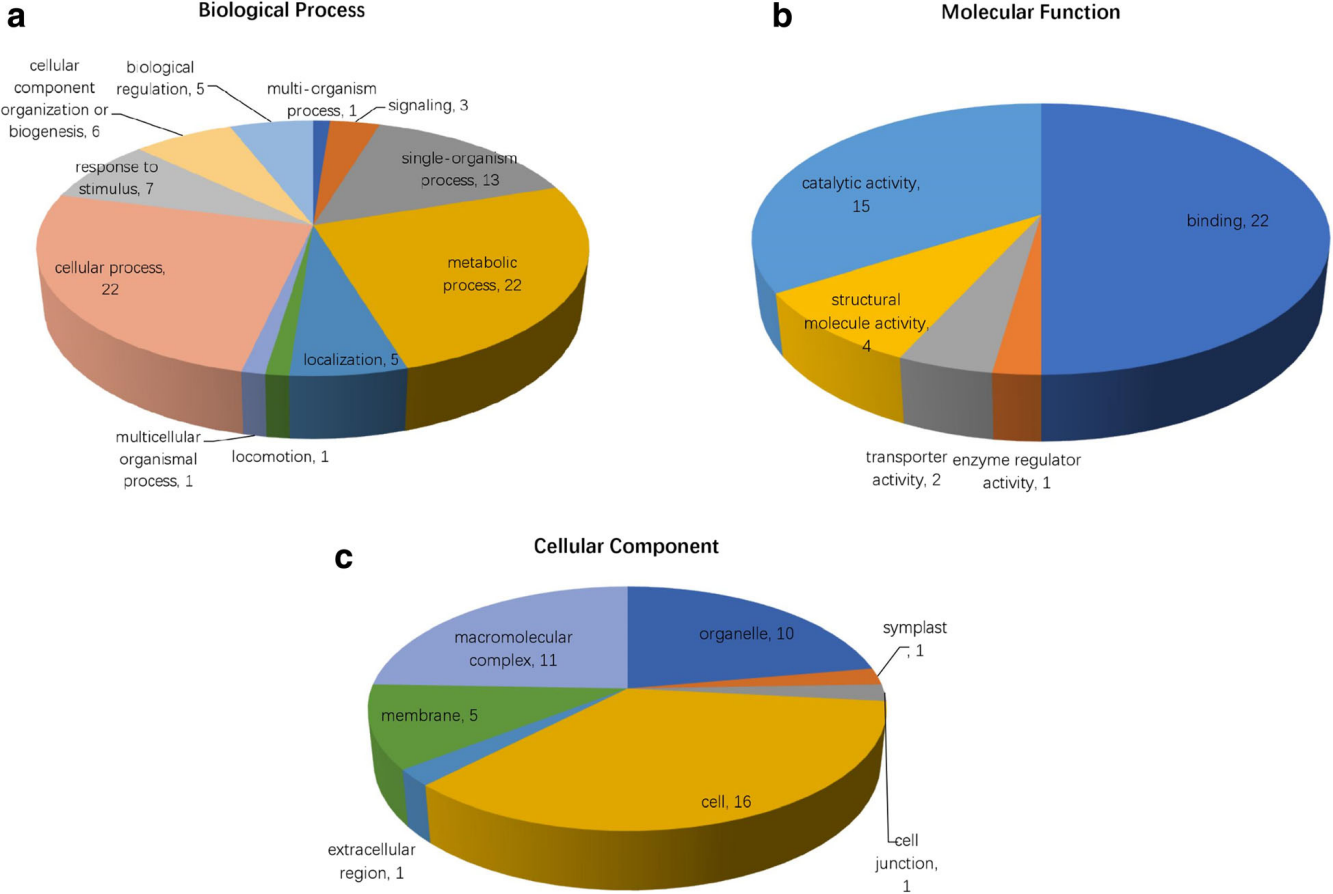
GO categories of the 35 proteins containing more than two unique peptide counts. The identified proteins were classified into biological process (**a**), molecular function (**b**) and cellular component (**c**) by WEGO according to their GO signatures. The number of proteins presented in the graph might exceed the total annotated proteins because some were grouped in more than one functional category

The 35 proteins with more than two unique peptide counts were analyzed using Gene Ontology (GO) and categorized into biological process, molecular function, and cellular component. The annotation by biological process (Fig. 4a) indicated that 22 proteins were related to cellular processes (GO 0009987), metabolic processes (GO 0008152), single-organism processes (GO 0044699), response to stimulus (GO 0050896), cellular component organization or biogenesis (GO 0071840), localization (GO 0051179) and biological regulation (GO 0065007). Other proteins were involved in multicellular organismal processes (GO 0032501), signaling (GO 0023052), multi-organism processes (GO 0051704) and locomotion (GO 0040011).

Five subcategories were assigned to molecular function (Fig. 4b). Binding activity (GO 0005488, 22 proteins, 62.86% of the 35 annotated peptides) and catalytic activity (GO 0003824, 15 proteins, 42.86% of the 35 annotated peptides) were the most representative molecular function categories. The remaining proteins were related to enzyme regulatory activity (GO 0030234), structural molecule activity (GO 0005198) and transport activity (GO 0005215).

The cellular components ontology (Fig. 4c) refers to the cellular regions where a gene product is active [18]. Following this category, of the identified proteins, 16 were present in the cell (GO 0005623), 11 in the macromolecular complex (GO 0032991), 10 in the organelle (GO 0043226), five in the membrane (GO 0016020), one in the extracellular region (GO 0005576), one in the symplast (GO 0055044) and one in cell junctions (GO 0030054).

Most cellular and metabolic processes were related to synthesis and degradation of macromolecules, particularly nucleotides, carbohydrates and proteins, and these processes might be associated with the invasion and development of *E. maxima*.

## Discussion

The coccidian parasite *E. maxima* is one of the seven *Eimeria* species which infect intestinal epithelial cells of chicken [19]. However, to date it is unclear why *E. maxima* preferentially invades and survives in jejunal epithelial cells of chicken small intestines. In the present study, the proteins of *E. maxima* sporozoites that bound to these epithelial cells were analyzed using the co-IP assay and QE shotgun LC-MS/MS analysis. Our results highlighted the importance of exploring the location specificity of *E. maxima*.

All invasive forms of apicomplexans, including *Eimeria* spp., possess a unique complex of organelles, the apical complex, located at the anterior end of the organism. The apical complex comprises rhoptries, micronemes, dense granules, as well as an apical assembly of cytoskeleton-associated structures including the conoid, polar/apical rings and micro tubular protrusions [20]. Most of these organelles secret proteins involved in parasite attachment, invasion and intercellular development [21, 22]. Microneme organelle proteins (MICs) are critical for parasite motility and host cell invasion [23]. MICs secreted in the early stages of invasion, participate in parasite attachment to the host cells and subsequent formation of the connection with the parasite actinomyosin system, thereby providing the platform from which an invasion will be initiated [24]. Previous studies proved that microneme secretion was rapidly increased when parasites contacted host cells and parasite invasion was blocked when microneme secretion was chemically inhibited [25, 26]. To date, nine microneme proteins have been reported in *Eimeria*: microneme proteins 1–7 (MIC 1-7) and apical membrane antigens 1 and 2 (AMA1 and 2) [27]. In recent studies, dual immunofluorescence staining of *E. tenella* microneme 3 (EtMIC3) and 5 (EtMIC5) on fixed and permeabilized sporozoites of *E. tenella* showed that the parasites were attached to host cells by the apical region, that EtMIC3 was located primarily at the apical tip of sporozoites [7], it could also bind to sialic acid-containing molecules on the epithelial cell surface of the hosts, and played a key role in cell invasion by sporozoites [7, 28]. Comparably, MIC1 and 13 of *T. gondii* (TgMIC1 and TgMIC13), and MIC1 of *Neospora caninum* (NcMIC1) showed similar binding capacity to sialic acid-containing molecules an indication of their roles in parasite-cell attachment regulation [28]. It has also been reported that 11 cysteine-rich receptor-like regions similar to the Apple domains of EtMIC5 were associated with parasite membrane and established an important correlation between cytoskeletal elements of parasites and receptors on the surface of host cells [29]. In the present study, three MICs (MIC2, MIC3 and MIC7) of *E. maxima* were identified. However, their roles during host cell attachment and invasion need to be deeply elucidated.

Several proteins associated with the sexual stage of *E. maxima*, including 56-, 82- and 230-kDa antigens, have been identified as potential vaccine targets for inducing transmission-blocking immunity, and these antigens were later proven to be 56-, 82- and 230-kDa gametocyte proteins [30]. Immunization with the 56- and 82-kDa gametocyte antigens promoted both cellular- and humoral-mediated immunity against experimental coccidiosis and reduced fecal oocyst shedding, which is an important component of the *Eimeria* life-cycle [19, 30]. In particular, Gam56 and Gam82 have been implicated in oocyst wall formation [31]. In this respect, the neutralization of Gam56 and Gam82 during the sexual stage could block the development of *E. maxima*. Previous studies reported that immunization with affinity-purified gametocyte antigens (APGA) from *E. maxima*, 56-, 82- and 230-kDa gametocyte proteins as subunit vaccines could induce partial protective immunity against *E. maxima*, *E. tenella* and *E. acervulina* infection [32]. In the present study, Gam56 and Gam82 were found among the proteins capable of binding to chicken jejunal epithelial cells. However, the role of gametocyte proteins in invasion needs to be better understood.

Protein 14-3-3 plays important roles in many regulatory processes including mitogenic signal transduction apoptotic cell death, cell cycle control and protein localization [33, 34]. Furthermore, *T. gondii* 14-3-3 protein has shown potentiality as a vaccine candidate against toxoplasmosis [35]. Moreover, other studies reported that 14-3-3 protein of *E. tenella* could interact with RNA-binding domain of telomerase reverse transcriptase [36]. In the present study, 14-3-3 protein of *E. maxima* bound to chicken jejunal epithelial cells, indicating the possible role that might be played by this protein during host cell invasion.

It is well known that all apicomplexan parasites share a unique host cell invasion mechanism that is dependent on actin [37, 38]. Actin filaments are composed of actin and other actin-binding proteins. These structures are highly dynamic in non-muscle cells and play critical roles in many cellular processes, including cell migration [39], cytokines and polarized growth [40, 41]. The actin cytoskeleton is involved in protein transportation in the early secretory pathway [42–44]. *Eimeria tenella* cannot enter host cells when the secreted proteins are neutralized by monoclonal antibodies [45], and/or when the cytoskeleton is blocked by drugs, such as cytochalasin D [46]. These results suggest that *E. tenella* motility during the invasion of host cells is an active process and depends on actin-based contractile systems regulated by different actin-binding proteins. In the present study, actin-like proteins were identified but their function in invasion needs to be further studied.

In the present study, several key enzymes that participate in energy and metabolic processes in *E. maxima* were found to bind to chicken jejunal epithelial cells, including lactate dehydrogenase (LDH), enolase 2 (ENO2) and aldolase (ALD). It has been reported that immunization with DNA vaccine carrying the *E. acervulina* LDH antigen gene could induce protective immunity against homologous infection and immunity could be enhanced by the co-expression of chicken IL-2 or IFN-γ [47]. Recent studies demonstrated that TgALD1 was required for energy production and was essential for efficient host cell invasion, based on its ability to bridge adhesin-cytoskeleton interactions in *T. gondii* [48, 49]. Another study evidenced that EtENO had a function not only in glycolysis during anaerobic intracellular stages but also in parasite invasion [50]. *Plasmodium falciparum* enolase was localized to the merozoite surface, indicating that PfENO might be involved in red blood cell invasion [51]. In this investigation, these proteins were found to interact with jejunal epithelial cells, but on the other hand, their exact during cell invasion by *E. maxima* need to be better studied.

In this study, 35 proteins binding to chicken jejunal epithelial cells contained more than two unique peptide counts, including heat-shock proteins 60, 70 and 90, elongation factor 1-alpha (EF-1α) and elongation factor 2. It was reported that EF-1α played an essential role in mediating host cell entry by the parasite and could be a candidate vaccine antigen [20]. Moreover, other studies indicated the heat shock protein 70 of *E. tenella* could stimulate the immune system and enhance the protective immunity elicited by *E. tenella* antigen microneme protein 2 (EtMIC2) against avian coccidiosis [52]. Additionally, heat-shock protein 90 was identified as crucial for *E. tenella* invasion and survival in host cells [53].

In the present study, Western blot analysis of proteins of co-cultured cells revealed that only four distinct protein bands were recognized using the antisera. However, in the analysis by QE shotgun LC-MS/MS, a total of 204 non-redundant proteins were identified by searching the *E. maxima* database. The difference between results obtained after using these two techniques could be explained based on their levels of sensitivity. Based on how QE shotgun LC-MS/MS works, this technique is more sensitive than conventional techniques such as Western blot.

## Conclusions

In conclusion, we found that 35 proteins of *E. maxima* with more than two unique peptide counts identified by shotgun LC-MS/MS could bind to chicken jejunal epithelial cells. The identified proteins were annotated according to Gene Ontology for molecular function, biological process and cellular localization. Many of these proteins play important roles in parasite invasion and motility. However, investigations on the molecular mechanisms of these proteins in the invasion process are warranted.

## Additional files

Additional file 1: **Table S1.** LC-MS/MS analysis of *E. maxima* sporozoite proteins binding to chicken jejunal epithelial cells. This file describes the details of the 204 non-redundant proteins that were identified using shotgun LC-MS/MS. (XLS 187 kb)

Additional file 2: **Table S2.** *Eimeria maxima* sporozoite soluble proteins binding to chicken jejunal epithelial cells with more than two unique peptide counts. This file describes the details of the 35 proteins binding to chicken jejunal epithelial cells that were identified with more than two unique peptide counts using shotgun LC-MS/MS. (XLS 95 kb)

## Abbreviations

ALD: aldolase
DAB: 3,3'-diaminobenzidine
ENO: enolase
GO: Gene Ontology
HBSS: Hanks’ balanced salt solution
HRP: horseradish peroxidase
LC-MS/MS: liquid chromatography and tandem mass spectrometry
LDH: lactate dehydrogenase
MDBK: Madin-Darby bovine kidney cells
MIC: microneme
PBS: phosphate-buffered saline
SDS-PAGE: sodium dodecyl sulfate polyacrylamide gel electrophoresis
